# Understanding the walking football population: sociodemographic, health, lifestyle, and participation insights from a national tournament cohort

**DOI:** 10.3389/fspor.2025.1744101

**Published:** 2026-01-09

**Authors:** Alfie G. Price, Bradley Sprouse, Philip J. Hennis, John Hough, Ali Ahmed, Thaila Hibberd, Ian Varley

**Affiliations:** 1 School of Science and Technology, Nottingham Trent University, Nottingham, United Kingdom; 2 University Hospitals of Derby and Burton NHS Foundation Trust, Derby, United Kingdom

**Keywords:** ageing, exercise, healthy ageing, physical activity, public health, team sports, walking football, walking soccer

## Abstract

**Aims:**

This study aimed to build a comprehensive understanding of who plays walking football and how participation relates to physical activity, well-being, and perceived health benefits, to assess its potential as a sustainable physical activity option for middle-aged and older adults.

**Methods:**

A cross-sectional, survey-based design examined the sociodemographic characteristics, health status, lifestyle behaviours, and participation experiences of 352 walking football players during The FA Walking Football Cup 2024 in England. Data were collected from six regional final events involving 84 teams.

**Results:**

Participants (mean age: 56 years; 55.3% men, 43.6% women) reported a broad age range (33–81 years) and diverse socioeconomic backgrounds (16.6% from the most deprived 30% of areas), but ethnic diversity was limited (95.3% White vs. 81.7% nationally). Compared to national averages, more participants had a healthy weight (men: 31.5% vs. 19%; women: 50.8% vs. 30%) and met the UK physical activity guideline of ≥150 min/week of moderate aerobic activity (75% vs. 63%). Despite 47% reporting health conditions, 70.4% experienced no limitations in daily activities. Mental well-being scores were higher, and stress and loneliness levels were lower than national averages. Over three-quarters of participants reported increased physical activity since starting walking football, with perceived improvements in social connections (82.6%), physical fitness (78.0%), and mental well-being (73.8%).

**Conclusion:**

Walking football attracts a broad player base and may support healthy ageing, even among those with chronic conditions. Greater efforts are needed to improve ethnic representation, but findings support its value as a health-enhancing physical activity option for middle-aged and older adults.

## Introduction

The ageing UK population and rising prevalence of multiple chronic conditions highlight the need for effective public health strategies focused on disease prevention, healthy ageing, and reducing inequalities (1). Physical activity is an effective tool in preventing and managing non-communicable diseases (2), yet over a third of middle-aged and older adults do not meet recommended guidelines of 150 min of moderate activity per week (3–5). Low activity levels contribute to functional decline (6), higher all-cause mortality risk (7), and substantial healthcare costs (8). Thus, there is a clear need for physical activity options that are accessible, inclusive, and socially engaging, with low barriers to entry and strong potential for sustained participation. Such interventions can play a vital role in promoting physical and mental well-being, strengthening social connections, and supporting wider public health goals for middle-aged and older adults.

Walking football has emerged as a promising form of physical activity tailored specifically to older populations. The sport modifies traditional association football rules by prohibiting running, limiting physical contact, and restricting ball height, creating a more accessible form of the game with a lower injury incidence than veteran's association football (9). Walking football provides opportunities for social interaction, camaraderie, and enjoyment (10), which are key determinants of exercise adherence and overall well-being (11). Emerging evidence also suggests this adapted sport may offer physiological health benefits (12), including improvements in blood glucose regulation and body composition (13, 14). Walking football could therefore serve as a viable means of engaging in physical activity, offering a more socially captivating and sport-based option compared to individual exercise (15–17).

Despite growing interest in walking football, little is known about those who play the sport, such as sociodemographic characteristics, physical and mental health, lifestyle behaviours, and whether it successfully reaches those who might benefit most from participation. The only known study consisted of three clubs in central Sweden, which found participants (*n* = 63) were aged 63–85 years old; approximately half had hypertension, nearly three-quarters used prescription medication, the majority were overweight, and average moderate-intensity physical activity was 53 min/day (18). Players attended organised sessions six times per month and had an average playing history of 3.2 years for men and 2.5 years for women (18). While informative, the study's small, geographically limited sample restricts the generalisability of findings. Such insights are valuable for considering the role of walking football as a sustainable and inclusive means of staying physically active in later life and informing strategies to enhance accessibility and retention.

Therefore, the aim of this study was to capture data from a large, geographically diverse cohort of walking football players to build a more comprehensive understanding of those who play the sport. By examining sociodemographic characteristics, health status, lifestyle behaviours, and participation experiences, we aimed to answer two primary questions: first, how does walking football participation relate to physical activity, well-being, and perceived health benefits; and second, who engages in walking football, and what are their profiles in terms of demographics, health, and lifestyle. Addressing these questions provides a cross-sectional perspective on walking football's role as a sustainable, inclusive physical activity option for middle-aged and older adults, especially those at risk of inactivity or chronic disease. The findings are intended to inform strategies to enhance accessibility, widen participation, and consider walking football's value as a health-enhancing activity. This information holds relevance for governing bodies, policymakers, and healthcare professionals interested in promoting sustainable forms of physical activity that support healthy ageing.

## Materials and methods

### Study design and setting

A cross-sectional cohort design examined participants in the six Regional Finals of The FA Walking Football Cup 2024, hosted by The Football Association of England (The FA). A cohort sampling approach was used, and no incentives or reimbursements were offered for participation. All measures were drawn from validated and reliable questionnaires, ensuring strong internal consistency and suitability for the study context. These standalone regional final events took place over four weeks (15/09/2024–13/10/2024) across six UK regions: South West (Devon), Midlands (Derby), North West (Wigan), North East (Sunderland), South (London), and South East (Essex). Teams qualified for the regional finals following success in local qualifier events, which took place across various regions of England through a total of 35 tournament days, mainly in held August 2024. A total of 84 teams participated across three categories: Women's 40 years+ (*n* = 33), Mixed Gender 50 years+ (*n* = 30), and Mixed Gender 60 years+ (*n* = 21). Participants were required to be registered players from these teams, with an estimated total player pool of 672 (based on an estimated eight players per team). The final sample size was determined by voluntary survey completion. Ethical approval was obtained by [redacted for anonymous manuscript], and informed consent was secured from all participants.

### Outcomes

Participants completed a structured survey covering multiple domains (Table 1; Supplementary Material S1).

**Table 1:** Overview of survey domains and corresponding items.

Domain	Items
Sociodemographic characteristics	Age, Ethnicity, Gender, Postcode (Socioeconomic Status), Sexual Orientation, Marital Status, Education, Employment Status
Health status	Body Mass Index, Chronic Diseases, Clinically Diagnosed Conditions, Impact of Long-Term Health Conditions on Daily Activities, Presence/Absence of Prescription Medication, Perceived Physical Health
Lifestyle behaviours	Sleep Quality (Sleep Condition Indicator: 0–8; higher values indicating better sleep), Smoking Status, Alcohol Consumption (Alcohol Use Disorders Identification Test-Consumption: 0–12; higher risk drinkers, ≥5)
Participation experiences	Duration of Overall Participation, Current Participation Levels, Participation in Other Sports, Perception of Intensity, Impact of Participation on Lifestyle, Enjoyment, Reasons for Participation, Exercise Habits Before and After Walking Football, Future Plans
Physical activity	Moderate-to-Vigorous Physical Activity Per Week (Exercise Vital Sign), Sitting Time Per Day (International Physical Activity Questionnaire—Short Form), Physical Activity History
Well-being	Mental Well-Being (Short Warwick-Edinburgh Mental Wellbeing Scale: 7–35; higher scores indicating better mental well-being), Stress levels (Perceived Stress Scale: 0–16; higher scores indicating higher perceived stress), Loneliness (Loneliness Scale: 3–9; higher scores indicating greater loneliness), Perceived Mental Health
Perceived health benefits	Perceived Changes in Aspects of Health

Sleep was assessed using the two-item Sleep Condition Indicator (19), a validated screening tool for insomnia (20). Smoking was measured through a single-item question: “*What is your current cigarette smoking behaviour (including hand-rolled cigarettes)?”* with response options: daily (≥1 cigarette/day), occasional (<1 cigarette/day), ex-smoker, and non-smoker. Alcohol consumption was measured using the three-item Alcohol Use Disorders Identification Test-Consumption (AUDIT-C) (21) where higher-risk drinkers were defined using an AUDIT-C cut-off of ≥5 which is a validated clinical screening tool (22).

Mental well-being was evaluated using the seven-item Short Warwick-Edinburgh Mental Wellbeing Scale (SWEMWBS) (23), stress via the four-item Perceived Stress Scale (PSS-4) (24), and loneliness with the three-item Loneliness Scale (25). Physical activity levels were measured using the three-item Exercise Vital Sign (EVS) (26), while sitting time was assessed with a single item from the International Physical Activity Questionnaire—Short Form (IPAQ-SF) (27). All tools are validated for use in adult UK or European populations (25, 26, 28–30).

Enjoyment of walking football was measured using the four-item Physical Activity Enjoyment Scale—Short Form (PACES-S) (31), validated in adult populations (32) and adapted for walking football (*“When I play walking football…”*). Additional survey items (Table 1; Supplementary Material S1) were assessed using a questionnaire adapted from Andersson et al. (18).

Postcode was used as an indirect indicator of socioeconomic status, with 181 participants providing valid data. Demographic characteristics of participants who provided postcodes did not differ substantially from those who did not.

Benchmarking comparisons from previous literature used to compare data from the studied cohort are descriptive and approximate, as methodological differences between studies, such as data-collection procedures, and reporting practices limit the precision of direct comparisons. Inferential analyses were not performed, as the aim was to provide descriptive profiling of the data while avoiding over-interpretation beyond what the sample could support.

Descriptive analyses were used in line with the study's aim to characterise the population and summarise key demographic and behavioural patterns.

## Results

### Sociodemographic characteristics

A total of 352 participants completed the survey (52.4% response rate from an estimated 672 players). A sample size of 245 was required for a 95% confidence level and 5% margin of error (33), indicating results are representative of the Regional Finals cohort. Ages (*n* = 318) ranged from 33 to 81 years (mean = 56 ± 7). Mean age was 53 (±7) for women (*n* = 141) and 59 mean (±6) for men (*n* = 172). Detailed sociodemographic characteristics of participants are presented in Table 2.

**Table 2:** Sociodemographic characteristics of walking football participants.

Sociodemographic characteristic	*N* (%) or mean ± SD
Gender (*n* = 349)	
Female	152 (43.6)
Male	193 (55.3)
Neither of the above/other	4 (1.1)
Age (y)	
Overall (*n* = 318)	56 ± 7
Women (*n* = 141)	52 ± 7
Men (*n* = 172)	59 ± 6
Ethnicity (*n* = 338)	
White	322 (95.3)
Mixed/multiple ethnic groups	8 (2.4)
Asian/Asian British	4 (1.2)
Black/Black British/Caribbean/African	3 (0.9)
Other Ethnic Groups	1 (0.3)
Geographic region (*n* = 352)	
South East	99 (28.1)
Midlands	71 (20.2)
South	57 (16.2)
South west	44 (12.5)
North west	41 (11.6)
North east	40 (11.4)
Socioeconomic status (*n* = 181)	
Most deprived 30% (1st–3rd deciles)	30 (16.6)
Middle 40% (4th–7th deciles)	74 (40.9)
Least deprived 30% (8th–10th deciles)	77 (42.5)
Sexual orientation (*n* = 340)	
Straight/heterosexual	300 (88.2)
Gay or lesbian	33 (9.7)
Other sexual orientation	2 (0.6)
Prefer not to say	5 (1.5)
Marital status (*n* = 342)	
Married or in a registered civil partnership	234 (68.4)
Singe and never married or in a registered civil partnership	48 (14.0)
Divorced or civil partnership dissolved	40 (11.7)
Separated	12 (3.5)
Widowed or surviving partner from a civil partnership	8 (2.3)
Educational attainment (*n* = 345)	
GCSEs, standard grades, or equivalent (e.g., O-levels, national 4/5)	108 (31.3)
Undergraduate degree or other degree level qualification (e.g., Bachelor's, Scottish Ordinary or Honours degree)	70 (20.3)
Postgraduate degree (e.g., Master's, PhD, postgraduate diploma or certificate)	54 (15.7)
A-levels, Scottish Highers, or equivalent (e.g., AS-levels, Advanced Highers, Welsh Baccalaureate)	41 (11.9)
Vocational or technical qualification (e.g., NVQ, SVQ, BTEC)	40 (11.6)
Apprenticeship	15 (4.3)
No formal qualifications	13 (3.8)
Other qualification	4 (1.2)
Employment status (*n* = 347)	
Employed full-time	159 (45.8)
Retired	98 (28.2)
Self-employed	52 (15.0)
Employed part-time	28 (8.1)
Unemployed	6 (1.7)
Unable to work	3 (0.9)
Other	1 (0.3)

### Health Status

Body mass index (BMI) data were available for 305 participants. The mean BMI was 26.8 kg/m^2^ [standard deviation (SD) = 4.7], placing the average participant in the overweight category. Moreover, 39.0% of participants had a BMI in the healthy range (18.5–24.99 kg/m^2^), 42.0% were overweight (25.0–29.99 kg/m^2^), 14.4% had obesity class I (30.0–34.99 kg/m^2^), 2.6% class II (35.0–39.99 kg/m^2^), and 2.0% class III (≥40.0 kg/m^2^).

**Figure 1:**
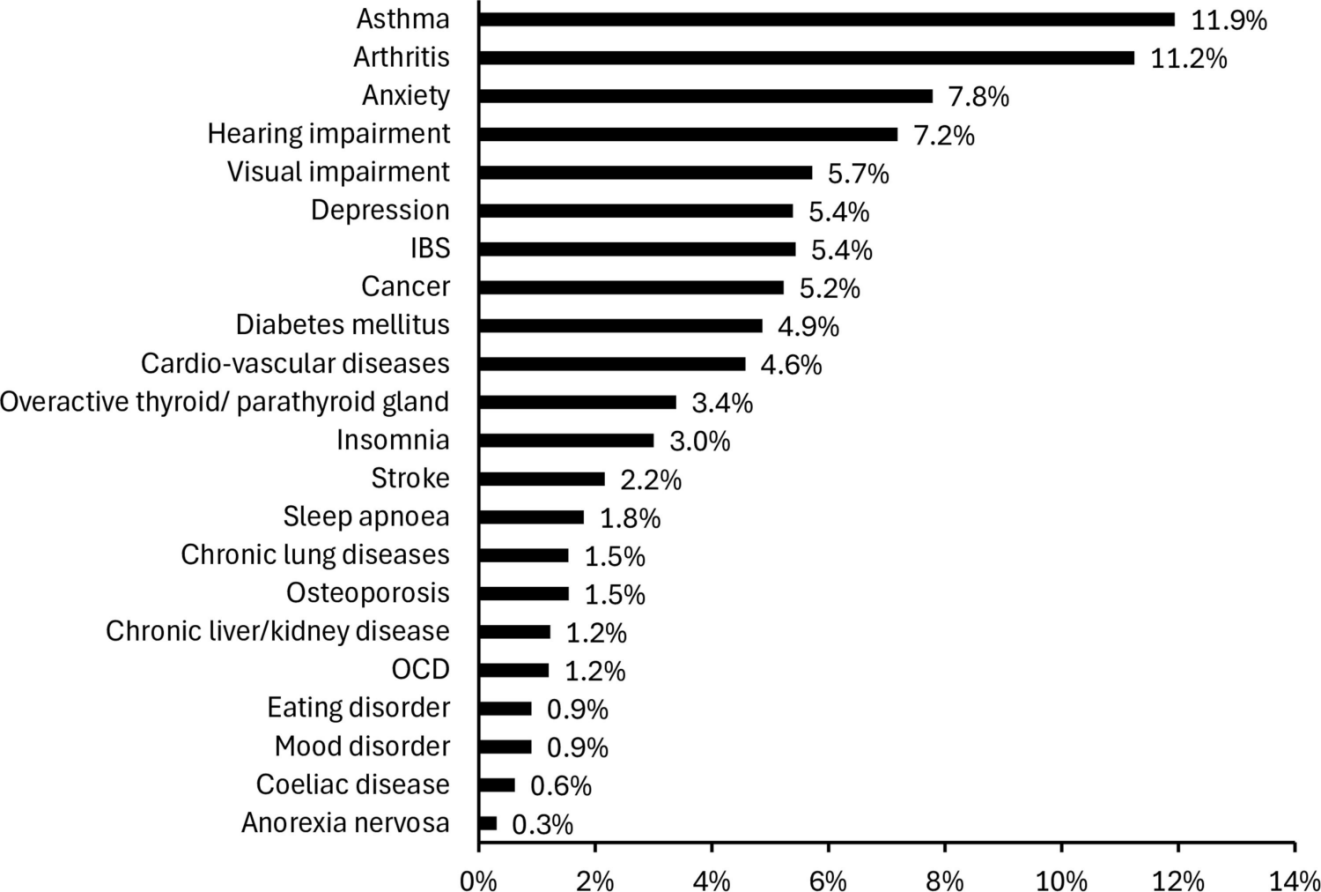
Self-reported health conditions among walking football participants.

Current physical health was described (*n* = 348) as very good (31.9%), good (56.0%), fair (11.5%), and poor (0.6%), with no participants reporting very poor health. Health conditions are presented in Figure 1, with 47% of respondents having at least one health condition. Regarding how much the long-term health condition(s) limits daily activities (*n* = 294), 70.4% reported no limitations, 23.8% a little bit, 3.7% somewhat limited, and 2.0% quite a bit or a lot limited. Regular prescription medication use was reported by 41.4% (*n* = 343).

### Lifestyle behaviours

Sleep quality (*n* = 334) had a mean score of 6.1 (SD = 2.1) and a median of 7 (IQR = 5–8). Of 345 respondents, 4.1% were daily smokers (≥1 cigarette per day), 1.7% occasional smokers (<1 cigarette per day), 10.7% ex-smokers, and 83.5% non-smokers. Alcohol consumption (*n* = 331) had a mean AUDIT-C score of 4.3 (SD = 2.9) and a median of 4 (IQR = 2–6). Table 3 compares these behaviours with national benchmarks, as well as well-being and physical activity.

**Table 3:** Summary of lifestyle behaviours, well-being, and physical activity levels compared to national/population averages.

Variable	Walking football cohort	National/population average	Source of benchmark
Sleep quality (0–8 with higher scores indicating better sleep)	Mean = 6.1	Not available nationally	–
Smoking status (daily/occasional)	5.8%	14.0% (aged 50–59)	ONS, (60)
Alcohol Consumption (higher risk drinkers, ≥5 AUDIT-C)	43.5%	45.8% (aged 18–92) 23.2% (average age 49.7 years)	Venturelli et al. (62) Mansfield et al. (61)
Mental well-being (7–35 with higher scores indicating better well-being)	Mean = 24.6	23.8 (aged 40–74)	NatCen Social Research et al. (41)
Stress (0–16 with higher scores indicating greater stress	Mean = 4.8	6.1 (aged 16–85)	Warttig et al. (30)
Loneliness (hardly ever/never lonely)	81.3%	67.3% (aged 50+)	ELSA Wave 8; cited in Age UK (42)
Physical activity (≥150 min per week at a moderate intensity)	75%	63% (aged 55–74)	Sport England (36)
Sitting Time (hours/day)	Mean = 5.7	9.5 for men, 9.0 for women (average age 47 years; accelerometer-based)	Hamer et al. (39)

ELSA, english longitudinal study of ageing wave 8. Extreme moderate-to-vigorous physical activity (MVPA) values (>1,050 min/week) were removed (*n* = 11) as per Kuntz et al. (34).

### Participation experiences

Among 339 respondents, walking football participation varied: <6 months (5.6%), 6–12 months (7.7%), 1–2 years (23.6%), 2–3 years (21.8%), 3–5 years (18.6%), and >5 years (22.7%). Participants (*n* = 328) averaged 7.1 sessions/month (SD = 3.7; media*n* = 6; IQR = 4–9; ∼1.6 sessions/week). Session duration (*n* = 326) averaged 67 min (SD = 18; median = 60; IQR = 60–60). Combining session frequency and duration (*n* = 326), participants averaged 469 min/month of walking football (SD = 259; ∼106 min/week). A total of 62.5% of respondents participated in other sports, averaging 6.7 sessions/month (SD = 4.9; median = 4; IQR = 4–8.5; ∼1.5 sessions/week) and 72 min/session (SD = 51; median = 60; IQR = 60–60; ∼108 min/week).

Enjoyment of walking football (*n* = 322) averaged 19.1 out of 20 (SD = 1.8; median = 20; IQR = 20–20). Primary motivations for participation (*n* = 330) included the desire to be part of a team or group (83.3%), to play football (83.0%), and to get exercise and stay fit (81.5%). Participants (*n* = 330) became aware of walking football through various sources: 26.1% via a local football club, 2.7% through a district or regional football association, 47.0% from a friend or family member, 22.1% via social media or other online platforms, 3.9% through media coverage (e.g., newspapers, TV), 2.1% via The FA's website or other official football websites, and 2.4% through a community event or local group. When participants (*n* = 339) were asked whether they plan to continue playing walking football in the long term (over the next year or more), 93.2% responded ‘Yes, definitely’, 6.5% said ‘Yes, likely’, 0.3% were unsure, and no participants selected ‘No, unlikely’ or ‘No, definitely not’.

Regarding perceived sufficiency of walking football to meet fitness goals among 337 participants, 37.1% responded ‘Yes, definitely’, 38.3% said ‘Yes, somewhat’, 9.8% were neutral or unsure, 13.4% said ‘No, not really’, and 1.5% responded ‘No, not at all’. The appropriateness of walking football intensity to one's fitness level was evaluated among 336 participants, with 69.6% considering it ‘perfect for my fitness level’, 11.3% finding it ‘somewhat challenging, but manageable’, 6.0% being neutral or unsure, 0.6% reporting it as ‘too intense’, and 12.5% considering it ‘not intense enough’. Of the 333 respondents to the statement “*Playing walking football has motivated me to adopt a healthier lifestyle in other areas.”*, 33.0% strongly agreed, 40.2% agreed, 24.0% were neutral, 1.8% disagreed, and 0.9% strongly disagreed.

### Physical activity

Extreme moderate-to-vigorous physical activity (MVPA) values (>1,050 min/week) were removed (*n* = 11) (34) from the MVPA-related analyses and retained for all other variables and analyses. Among 324 respondents, average MVPA was 270 min/week (SD = 186; median = 231; IQR = 148–360), with 75% meeting ≥150 min/week. Sitting time (*n* = 318) averaged 5.7 h/day (SD = 7.2; median = 4.8; IQR = 3–6).

Physical activity history is presented in Table 4 in response to the question “*How often did/do you take part in sports and leisure time exercise? (e.g., running, racquet sports, football, rugby, hockey, dancing etc). Please tick your best approximation for each age category”.*

**Table 4:** Self-reported frequency of sports and leisure time exercise across age categories.

Age Category	None	Occasionally (once a month)	Frequently (once a week)	Very frequently (more than once a week)
Up to the age of 18 (*n* = 316)	4.4%	5.7%	13.3%	76.6%
When you were aged 18–29 (*n* = 313)	4.8%	9.3%	22.0%	63.9%
When you were aged 30–49 (*n* = 318)	5.0%	13.8%	29.6%	51.6%
Since you were aged 50 (*n* = 276)	1.1%	2.9%	29.7%	66.3%

Assessment of physical activity levels in the 5–10 years before starting walking football (*n* = 332) indicated that 27.7% were ‘very active’, 50.0% ‘moderately active’, 17.8% ‘lightly active’, and 4.5% ‘sedentary’. Changes in physical activity levels since starting walking football (*n* = 330) revealed 37.9% experienced a significant increase, 39.7% a slight increase, 20.3% no change, 2.1% a slight decrease, and 0% a significant decrease.

### Well-being

Mental well-being (*n* = 332) had a mean score of 24.6 (SD = 4.9), while perceived stress (*n* = 336) averaged 4.8 (SD = 3.0). Loneliness data (*n* = 342) revealed a mean score of 3.6 (SD = 1.2) and showed that 81.3% reported being ‘hardly ever or never lonely’, 17.5% felt ‘lonely some of the time’, and 1.2% reported feeling ‘often lonely’. Among those who rated their mental health (*n* = 346), 42.5% described it as very good, 45.1% good, 11.3% fair, 0.9% poor, and 0.3% very poor.

### Perceived health benefits

**Figure 2:**
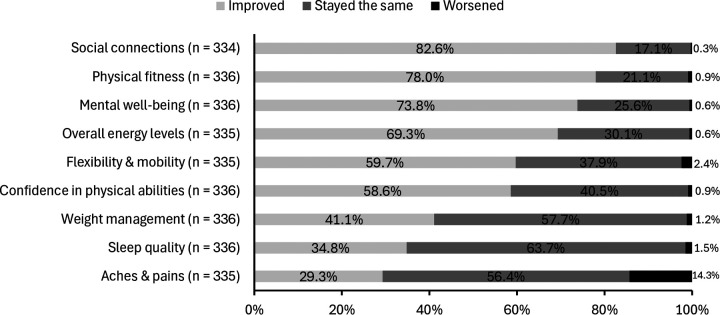
Perceived impact of walking football participation on health dimensions.

Perceived health benefits of walking football were assessed through multiple dimensions. Figure 2 presents responses to the question: “*How has participating in walking football affected the following aspects of your health”*, whereby the greatest proportion of participants reported perceived improvements in social connections, physical fitness, and mental well-being.

## Discussion

This study examined two primary questions: (1) how does walking football participation relate to physical activity levels, well-being, and perceived health benefits; and (2) who engages in walking football, in terms of their demographics, health, and lifestyle. To explore these questions, we surveyed 352 walking football players at The FA Walking Football Cup 2024 in England and found that 47% reported living with a health condition, yet 70.4% of those experienced no limitations in daily activities. Mental well-being scores were higher, stress levels lower, and loneliness less prevalent than national averages, suggesting potential psychosocial benefits of participation. Three-quarters met or exceeded the UK CMO and WHO guidelines of ≥150 min/week of moderate aerobic activity (vs. 63% nationally for adults aged 55–74), and 77.6% indicated they became more physically active since starting walking football. Participants also reported wide-ranging perceived benefits, including improved social connections (82.6%), physical fitness (78.0%), and mental well-being (73.8%). Nearly all (99.7%) intended to continue playing over the next year, pointing to strong long-term engagement. These findings provide valuable insights into walking football players competing in a national tournament and highlight walking football's inclusivity and sustainability, as well as its potential physical and mental health impacts in this population.

Research Question 1: How does walking football participation relate to physical activity, well-being, and perceived health benefits?

### Physical activity and walking football participation

Participants in the present study averaged 270 min/week of MVPA, exceeding the UK CMO and WHO guidelines of ≥150 min (4, 5). While national data show an average of 419 min/week in adults aged 55–64 years, these estimates include a broad range of activities and are based on telephone interviews (35). Notably, 75% of walking football players met the ≥150 min/week guideline, compared to 63% of adults aged 55–74 years nationally (36), suggesting they are a particularly active cohort. Swedish players recorded 53.2 min/day of moderate activity via accelerometer, supporting this pattern (18). However, our findings extend these results by demonstrating high physical activity levels across a larger and more geographically diverse English sample, spanning a wider age range. Participants in the present study averaged 1.6 walking football sessions per week (67 min/session), equating to 106 min/week and highlighting its major, but not exclusive, contribution to overall activity levels. Furthermore, 66.3% reported engaging in ‘very frequent’ (more than once a week) physical activity since age 50, compared to 51.6% when aged 30–49, highlighting the sport's potential to support sustained activity in later life. Walking football appears highly sustainable, with 86.7% of participants engaged for over one year and 99.7% intending to continue. This may be explained by the high enjoyment levels (mean PACES-S score = 19.11 out of 20), indicating that walking football is a highly engaging activity. Enjoyment is a key predictor of exercise adherence (37, 38), reinforcing the value of walking football as a sustainable exercise option.

A notable proportion of participants (37.9%) reported a significant increase in activity since joining, and a similar proportion reported a slight increase, suggesting that for many, walking football serves as a catalyst for greater physical activity engagement. Indeed, 62.5% reported engaging in other sports, with a mean participation rate of 1.5 sessions and 108 min per week, similar to levels of participation in walking football. Additionally, the mean self-reported sitting time of 5.7 h per day is relatively low, reinforcing the active lifestyle of participants. This contrasts with the average sitting time of more than 9 h per day measured using accelerometery in a large sample of British adults (39). However, self-reporting has been shown to underestimate sedentary behaviour (40), and accelerometer-based sedentary time in a cohort of Swedish walking football players revealed an average of 8 h per day (18).

### Well-being and perceived health benefits

Mental well-being scores for walking football players (24.6) were higher than the national average in 2019 for adults aged 40–74 years old (23.8) (41), although this comparison should be interpreted cautiously as the national figures were collected using a different sampling frame and survey methodology. Stress levels were lower than normative data from a non-clinical English adult population (4.8 vs. 6.1) (30). Additionally, loneliness levels were lower, with 81.3% of participants reporting they ‘hardly ever or never’ felt lonely, compared to 67.3% among people aged 50 and over in England (English Longitudinal Study of Ageing Wave 8, 2016–2017; cited in (42). While causality cannot be established, these findings suggest that walking football may contribute to enhanced mental well-being and reduced social isolation. Indeed, 73.8% reported improved mental well-being, and 82.6% enhanced social connections, aligning with existing literature suggesting that walking football may support mental health (43, 44). Self-rated mental health was high, with 87.6% reporting ‘good’ or ‘very good’ mental health, similar to findings from a Swedish cohort (18). Our study supports these positive mental health outcomes across a broader demographic, offering further evidence that walking football may provide mental health benefits for diverse participants. These benefits may, in part, stem from the team-based nature of walking football which fosters a sense of belonging and purpose (45). Evidence suggests that sport-based physical activity offers greater social connection than individual exercise formats like spinning and resistance training (15–17), and the present study identified ‘being part of a team or group’ as the leading motivation for participation.

Walking Sports as a Mechanism for Promoting Health and Well-BeingRecent evidence from other walking sports supports the present findings in walking football. Research on walking netball demonstrates that adapted walking formats are acceptable, feasible, and effective for increasing physical activity, particularly when delivery is tailored to the population (46). In addition, older adults engaging in walking sports have reported higher self-rated health and physical activity levels than non-participants, reinforcing the value of accessible sport formats for those facing barriers to traditional sport (47). Data also show that enjoyment in walking sports is predicted by factors such as intrinsic motivation, highlighting the importance of socially connected and meaningful participation opportunities (48). Together, these studies align with current findings by showing that walking football, and other walking-based sports, may offer a health-enhancing, enjoyable and inclusive way for older adults to remain physically active.

Research Question 2: Who engages in walking football and what are their sociodemographic characteristics, health status, and lifestyle behaviours?

### Sociodemographic characteristics

Our findings demonstrate that walking football players span a wide range of ages (33–81 years), suggesting the sport is accessible to a broad age demographic. Attracting older adults is an encouraging sign of inclusivity because sport participation typically declines with age (49–51). Indeed, over two-thirds of respondents believed the intensity of walking football was appropriate for their fitness level, and three-quarters believed walking football was sufficient to meet fitness goals, suggesting the sport effectively caters to its target demographic.

A striking lack of ethnic diversity was observed in the walking football cohort, with 95.3% of participants identifying as White, significantly higher than the national average of 81.7% for England and Wales (52). Participation among Black (0.9% vs. 4.0%) and Asian (1.2% vs. 9.3%) individuals was markedly underrepresented (52). These comparisons should be interpreted cautiously as the national figures are derived from census data collected using different sampling and response methods than those used in this study. Nevertheless, this disparity underscores a critical equity gap and highlights the urgent need for inclusive outreach and culturally tailored initiatives to broaden participation among ethnically diverse communities. Awareness of walking football was primarily driven by word-of-mouth (47.0%), suggesting that social networks play a crucial role in recruitment. In contrast, social media made up 22.1% of awareness, aligning with findings from Sweden (27.8%) (18) and suggesting capacity for greater media and online coverage to increase participation.

Socioeconomic status findings indicate a broad representation across deprivation levels, with 16.6% of participants residing in the most deprived areas. This suggests that walking football successfully reaches individuals across different socioeconomic backgrounds in England. The study also revealed a higher proportion of gay and lesbian participants (9.7%) compared to the national proportion of 1.5% (53), indicating that walking football provides an inclusive environment for LGBTQ+ individuals. The diverse range of marital statuses, educational attainments, and employment statuses further underscores the sport's broad appeal and capacity to facilitate social connections among varied demographics.

### Health Status

A greater proportion of walking football players self-reported a healthy weight profile (BMI 18.5–24.99 kg/m^2^) than national averages (male players: 31.5% vs. 19%–21% of men aged 55–74; female players: 50.8% vs. 30%–35% of women aged 45–64 (54). Obesity rates were also lower among walking football players: 17.1% of men and 21.3% of women, vs. 28% and 30% nationally (54). These findings suggest walking football may support weight management in middle-aged and older adults.

Furthermore, a significant proportion (47%) of participants reported at least one health condition (Figure 1), yet 70.4% of those reported no limitations in daily activities. This suggests walking football may help overcome health-related barriers to sport participation in middle-aged and older adults (55–57). Self-rated physical health was high (87.9% ‘good’ or ‘very good’), comparable to Swedish walking football players (84.9% ‘good’, ‘very good’, or ‘excellent’) (18) and thereby reinforcing these results in a broader participant sample. In contrast, regular use of prescription medication was lower than in the Swedish cohort (41.4% vs. 73.6%), likely reflecting a younger sample (mean age 56 vs. 71 years) (18). These findings demonstrate that individuals managing chronic conditions feel comfortable participating in walking football.

### Lifestyle behaviours

The average sleep quality score among participants was 6.1 (on a scale of 0–8 and higher values indicating better sleep), suggesting good overall sleep health. Given that regular moderate-intensity physical activity improves sleep quality (58), it is plausible that participation in walking football contributes positively to sleep patterns. Additionally, smoking rates among walking football players were lower than national averages (5.8% vs. 11.6% overall, 14.0% among those aged 50–59 and 7.6% for those aged 60+) (59, 60). This suggests that walking football players may be more health-conscious than the general population, or that participation in the sport is associated with positive behavioural changes.

Drinking behaviour revealed that 56.5% of participants were classified as low-risk drinkers (scores of 0–4), compared to 76.8% in a general UK primary care sample (61). Conversely, a higher proportion of walking football players screened as higher risk drinkers (scores of 5–12) than in the general population (43.5% vs. 23.2%) (61). However, the population used by Mansfield et al. (61) is a UK primary care sample, which may not be fully representative of the general population. The present study's findings aligned more closely with findings from a smaller UK sample (45.8% in a North London dental patient study) (62). Further research is needed to explore whether walking football participation influences drinking behaviours or whether other lifestyle factors contribute to these findings. Encouragingly, 73.2% of participants agreed that playing walking football had motivated them to adopt a healthier lifestyle in other areas, highlighting its potential as a gateway to broader positive health behaviours.

### Strengths and limitations

A key strength of this study lies in its large sample and wide geographic distribution of respondents, spanning six different regions of England, which enhances the generalisability of the findings. The breadth of variables collected provides a comprehensive profiling of walking football players, and the inclusion of psychometrically validated questionnaires strengthens the reliability of the self-reported data. However, several limitations should be acknowledged. The cross-sectional design prevents causal inferences regarding the effects of walking football participation, and self-reported data may be susceptible to recall bias and social desirability effects, particularly in sensitive domains like mental well-being and loneliness. Furthermore, the sample was drawn from tournament attendees, who may represent a particularly active and motivated group of walking football players, potentially limiting generalisability to less competitive players. Indeed, participants were those who qualified from the Local Qualifying Round of The FA Walking Football Cup 2024 and entered the Regional Finals, therefore likely representing more skilled walking football players who could be a fitter, more health-conscious, and longer-engaged subset of the broader walking football population, which should be considered when interpreting the findings and not generalised of all walking football programmes or standards of play. Finally, although minimum age thresholds were in place for each tournament category, two participants who provided age data reported being younger than the eligibility criteria. It is therefore possible that a small number of responses came from individuals not eligible to participate in the tournament. Future research should consider longitudinal or controlled intervention designs to assess causal effects, and include non-tournament, community-level samples and more ethnically diverse settings, supported by partnerships with community organisations and culturally tailored outreach, to address the participation gaps identified.

## Conclusion

This study provides novel insights into the sociodemographic characteristics, health status, lifestyle behaviours, and participation experiences of walking football players in England. Participants spanned a broad spectrum of socioeconomic, educational, and employment backgrounds, and displayed favourable health and lifestyle indicators, including high physical activity levels, good mental well-being, and low loneliness levels. The high prevalence of chronic conditions alongside strong long-term participation intentions highlights walking football's potential to promote sustained physical activity even among those with long-term health issues. These findings reinforce the significant promise of walking football as a catalyst for healthy ageing. With its ability to enhance physical health, mental well-being, and social connection, walking football should be actively championed by governing bodies, policymakers, and healthcare professionals as a valuable tool in addressing the challenges of an ageing population and promoting long-term public health. Nonetheless, the limited ethnic diversity observed within the current participant base signals an urgent need to improve inclusivity and access for underrepresented communities.

## Data Availability

The original contributions presented in the study are included in the article/Supplementary Material, further inquiries can be directed to the corresponding author.
