# Supplementary material for: Understanding the walking football population: sociodemographic, health, lifestyle, and participation insights from a national tournament cohort
Source: Front Sports Act Living. 2026 Jan 9;7:1744101. doi: 10.3389/fspor.2025.1744101 (PMC12827653; doi:10.3389/fspor.2025.1744101)
Supplement: Supplementary file 1 [file Datasheet1.pdf]

1.1 What is your age (years)?

____

1.2 How would you describe your ethnicity?

White

- ☐ English / Welsh / Scottish / Northern Irish / British
- ☐ Irish
- ☐ Gypsy or Irish Traveller
- ☐ Roma
- ☐ Other White

Mixed / Multiple ethnic groups

- ☐ White and Black Caribbean
- ☐ White and Black African
- ☐ White and Asian
- ☐ Other Mixed

Asian / Asian British

- ☐ Indian
- ☐ Pakistani
- ☐ Bangladeshi
- ☐ Chinese
- ☐ Other Asian

Black / Black British / Caribbean / African

- ☐ Caribbean
- ☐ African
- ☐ Other Black

Other ethnic group

- ☐ Arab
- ☐ Any other ethnic group

1.3 What is your gender?

☐ Male ☐ Female ☐ Trans male

☐ Trans female ☐ Non-binary ☐ Prefer not to say

1.4 What is your postcode?

____

1.5 Which of the following best describes your sexual orientation?

☐ Straight/Heterosexual ☐ Gay or Lesbian

☐ Bisexual ☐ Other sexual orientation

☐ Prefer not to say

1.6 What is your height? (Please skip if unsure)

☐.☐☐ metres OR ☐ feet ☐ inches

1.7 What is your weight? (Please skip if unsure)

☐☐ kilograms OR ☐ stone ☐ pounds

1.8 What is your legal marital or registered civil partnership status?

☐ Single, never married or in a registered civil partnership

☐ Married or in a registered civil partnership

☐ Separated

☐ Divorced or civil partnership dissolved

☐ Widowed or surviving partner from a civil partnership

1.9 What is the highest level of education you have completed?

☐ No formal qualifications

☐ GCSEs, Standard Grades, or equivalent (e.g. O-levels, National 4/5)

☐ A-levels, Scottish Highers, or equivalent (e.g. AS-levels, Advanced Highers, Welsh Baccalaureate)

☐ Apprenticeship

☐ Vocational or technical qualification (e.g. NVQ, SVQ, BTEC)

☐ Undergraduate degree or other degree level qualification (e.g. Bachelor's, Scottish Ordinary or Honours degree)

☐ Postgraduate degree (e.g. Master's, PhD, postgraduate diploma or certificate)

☐ Other qualification (please specify):___________________________________

1.10 What is your current employment status?

☐ Employed full-time

☐ Employed part-time

☐ Self-employed

☐ Unemployed

☐ Retired (whether receiving a pension or not)

☐ Unable to work due to disability or illness

☐ None of the above (please specify):_______________________________________

Your Health

2.1 How would you describe your current physical health?

☐ Very good ☐ Good ☐ Fair ☐ Poor ☐ Very poor

2.2 How would you describe your current mental health?

☐ Very good ☐ Good ☐ Fair ☐ Poor ☐ Very poor

2.3 Do you regularly take any prescription drugs for a medical condition?

☐ Yes ☐ No

2.4 Do you have, or have you ever had any of the following diseases? (If yes, please specify which disease or type)

1.	Chronic lung diseases	Yes ☐	No ☐	Type____________
2.	Cardio-vascular diseases	Yes ☐	No ☐	Type____________
3.	Peripheral arterial disease	Yes ☐	No ☐	Type____________
4.	Diabetes mellitus	Yes ☐	No ☐	Type____________
5.	Stroke	Yes ☐	No ☐	Type____________
6.	Cancer	Yes ☐	No ☐	Type____________
7.	Osteoporosis	Yes ☐	No ☐	Type____________
8.	Arthritis (Rheumatoid/Osteo)	Yes ☐	No ☐	Type____________
9.	Chronic liver or kidney disease	Yes ☐	No ☐	Type____________
10.	Anorexia nervosa	Yes ☐	No ☐	Type____________
11.	Overactive thyroid/ parathyroid gland	Yes ☐	No ☐	Type____________
12.	Coeliac disease or malabsorption	Yes ☐	No ☐	Type____________

Other disease (please specify):____

2.5 Do you have any of the following clinically diagnosed conditions?

1.	Anxiety	Yes ☐	No ☐
2.	Depression	Yes ☐	No ☐
3.	Asthma	Yes ☐	No ☐
4.	Insomnia	Yes ☐	No ☐
5.	Sleep apnoea	Yes ☐	No ☐

(Please turn over)

6. Obsessive compulsive disorder (OCD) Yes ☐ No ☐

7. Mood disorder (e.g. Bipolar) Yes ☐ No ☐

8. Schizophrenia Yes ☐ No ☐

9. Eating disorder Yes ☐ No ☐

10. Irritable bowel syndrome (IBS) Yes ☐ No ☐

11. Visual impairment Yes ☐ No ☐

12. Hearing impairment Yes ☐ No ☐

Other condition (please specify):____

2.6 Please state how much your long-term health problem(s) limit your daily activities? (Skip if not applicable)

☐ Not at all ☐ A little ☐ Somewhat ☐ Quite a bit ☐ A lot

Sleeping Behaviour

3.1 Thinking about the past month, to what extent has poor sleep troubled you in general?

☐ Not at all ☐ A little ☐ Somewhat ☐ Much ☐ Very much

3.2 Thinking about a typical night in the last month, how many nights a week do you have a problem with your sleep?

☐ 0-1 ☐ 2 ☐ 3 ☐ 4 ☐ 5-7

Smoking Behaviour

4.1 What is your current cigarette smoking behaviour (including hand-rolled cigarettes)?

☐ Daily smoker (at least one cigarette per day)

☐ Occasional smoker (less than one cigarette per day)

☐ Ex-smoker

☐ Non-smoker

Drinking Behaviour

Alcohol unit reference

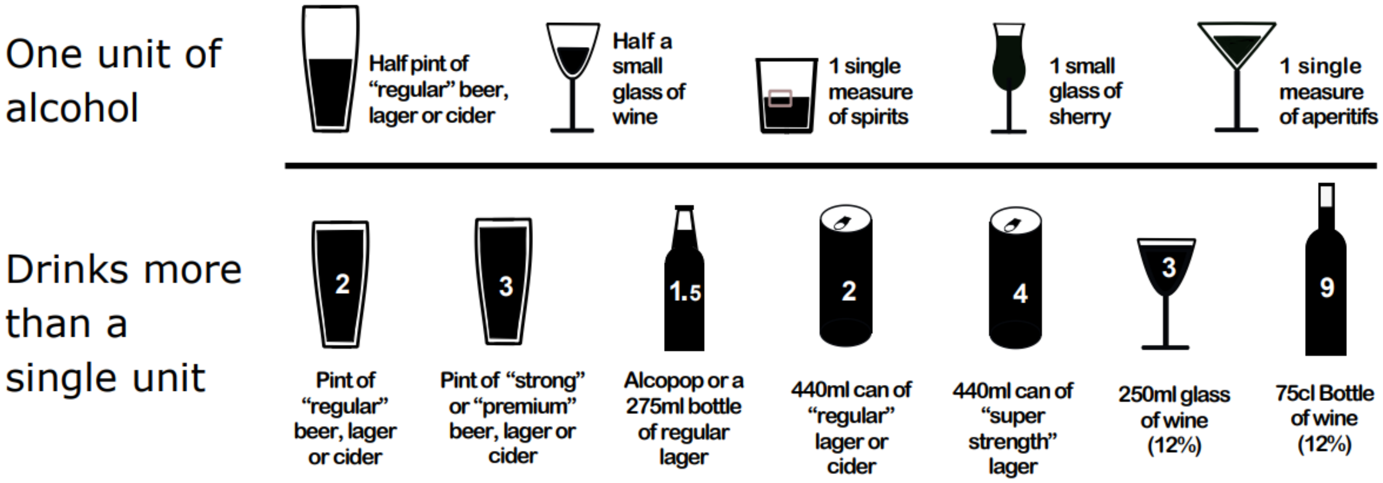

5.1 How often do you have a drink containing alcohol?

☐ Never

☐ Monthly or less

☐ 2 to 4 times per month

☐ 2 to 3 times per week

☐ 4 or more times per week

5.2 How many units of alcohol do you drink on a typical day when you are drinking?

☐ 0 to 2

☐ 3 to 4

☐ 5 to 6

☐ 7 to 9

☐ 10 or more

5.3 How often have you had 6 or more units of alcohol if female, or 8 or more if male, on a single occasion in the last year?

☐ Never

☐ Less than monthly

☐ Monthly

☐ Weekly

☐ Daily or almost daily

Mental Well-being

6.1 The following statements are about feelings and thoughts. Please tick one box per row that best describes your experience of each statement over **the last 2 weeks**.

	None of the time	Rarely	Some of the time	Often	All of the time
1. I've been feeling optimistic about the future	☐	☐	☐	☐	☐
2. I've been feeling useful	☐	☐	☐	☐	☐
3. I've been feeling relaxed	☐	☐	☐	☐	☐
4. I've been dealing with problems well	☐	☐	☐	☐	☐
5. I've been thinking clearly	☐	☐	☐	☐	☐
6. I've been feeling close to other people	☐	☐	☐	☐	☐
7. I've been able to make up my own mind about things	☐	☐	☐	☐	☐

Perceived Stress

7.1 The questions in this scale are about your feelings and thoughts during **the last month**. Please tick one box per row that best describes **how often** you felt or thought a certain way.

	Never	Almost never	Sometimes	Fairly often	Very often
1. In the last month, how often have you felt that you were unable to control the important things in your life?	☐	☐	☐	☐	☐
2. In the last month, how often have you felt confident about your ability to handle your personal problems?	☐	☐	☐	☐	☐
3. In the last month, how often have you felt that things were going your way?	☐	☐	☐	☐	☐
4. In the last month, how often have you felt difficulties were piling up so high that you could not overcome them?	☐	☐	☐	☐	☐

Loneliness

8.1 How often do you feel that you lack companionship?

☐ Hardly ever ☐ Some of the time ☐ Often

8.2 How often do you feel left out?

☐ Hardly ever ☐ Some of the time ☐ Often

8.3 How often do you feel isolated from others?

☐ Hardly ever ☐ Some of the time ☐ Often

Physical Activity Behaviour

9.1 On average, how many days per week do you engage in moderate to strenuous exercise (like a brisk walk)?

_____ **days**

9.2 On average, how many minutes per day do you engage in exercise at this level?

_____ **minutes**

9.3 How many days a week do you perform muscle strengthening exercises, such as bodyweight exercises or resistance training?

_____ **days**

The following question is about the time you spent **sitting** on weekdays during the **last 7 days**. Include time spent at work, at home, while doing course work and during leisure time. This may include time spent sitting at a desk, visiting friends, reading, or sitting or lying down to watch television.

9.4 During the **last 7 days**, how much time did you spend **sitting** on a **week day**?

_____ **hours per day**

_____ **minutes per day**

Past Physical Activity

10.1 How often did/do you take part in sports and leisure time exercise? (e.g. running, racquet sports, football, rugby, hockey, dancing etc).

Please tick your best approximation for each age category.

	None	Occasionally (once a month)	Frequently (once a week)	Very frequently (more than once a week)
Up to the age of 18	☐	☐	☐	☐
When you were aged 18-29	☐	☐	☐	☐
When you were aged 30-49	☐	☐	☐	☐
Since you were 50	☐	☐	☐	☐

Your Experience With Walking Football

11.1 How long have you been participating in walking football?

☐ Less than 6 months ☐ 6 months to 1 year

☐ 1 to 2 years ☐ 2 to 3 years

☐ 3 to 5 years ☐ More than 5 years

11.2 How many times a **month** do you participate in walking football?

_____ **times per month**

11.3 How long is a typical walking football session?

_____ **minutes per session**

11.4 How many times a **month** do you participate in other sports?

_____ **times per month**

11.5 How long does a typical session last for the other sports you participate in?

_____ **minutes per session**

11.6 How has participating in walking football affected the following aspects of your health? Please tick one box per row.

	Worsened	Stayed the same	Improved
Aches & pains (e.g., joints, muscles)	☐	☐	☐
Physical fitness	☐	☐	☐
Flexibility & mobility	☐	☐	☐
Overall energy levels	☐	☐	☐
Mental well-being (e.g., mood, stress)	☐	☐	☐
Sleep quality	☐	☐	☐
Social connections	☐	☐	☐
Weight management	☐	☐	☐
Confidence in physical abilities	☐	☐	☐

11.7 In relation to the previous question, have you made any changes to your participation (e.g., frequency, intensity) due to these effects?

☐ Yes ☐ No

If Yes, please describe:____

____

11.8 Do you feel that playing walking football provides you with enough exercise to meet your fitness goals?

☐ Yes, definitely

☐ Yes, somewhat

☐ Neutral/Unsure

☐ No, not really

☐ No, not at all

11.9 Do you feel that the intensity of walking football is appropriate for your fitness level?

☐ Yes, it's perfect for my fitness level

☐ It's somewhat challenging, but manageable

☐ Neutral/Unsure

☐ It's too intense for my fitness level

☐ It's not intense enough for my fitness level

11.10 To what extent do you agree with the following statement: *"Playing walking football has motivated me to adopt a healthier lifestyle in other areas."*

☐ Strongly agree

☐ Agree

☐ Neither agree nor disagree

☐ Disagree

☐ Strongly disagree

11.11 Have you suffered an injury when participating in walking football?

☐ Yes ☐ No

If Yes, please provide a brief description of the following.

*Type of injury:*____

*Body part:*____

*Duration of recovery (in days/weeks/months):*_________________________________

If Yes, have you ever suffered a **similar** injury in any other sport or physical activity throughout your life?

☐ Yes ☐ No

11.12 Have you ever suffered an injury when participating in recreational sports (not related to walking football)?

☐ Yes ☐ No ☐ I have never participated in any other sport

Enjoyment

12.1 Please tick one box per row that best describes your experience of each statement.
"When I play walking football…"

	Strongly disagree	Disagree	Neutral	Agree	Strongly agree
1. I enjoy it	☐	☐	☐	☐	☐
2. I find it pleasurable	☐	☐	☐	☐	☐
3. It is very pleasant	☐	☐	☐	☐	☐
4. It feels good	☐	☐	☐	☐	☐

12.2 What are your main reasons for participating in walking football? Tick all that apply.

☐ I used to play football and miss it

☐ To play at a level suited to my ability and ambition

☐ To be part of a team or group

☐ To socialise and meet others

☐ To play football

☐ For the "locker room" experience and banter

☐ To get exercise and stay fit

☐ To spend time outdoors

☐ To compete with others

☐ To lose weight

☐ I can't play other sports due to previous injury or illness

☐ To prove to others that I can still play

☐ For fun

☐ It boosts my confidence

Other:____

Before Walking Football

13.1 Before starting walking football (over the past 5-10 years), how physically active were you?

☐ Very active ☐ Moderately active ☐ Lightly active ☐ Sedentary

13.2 Since starting walking football, how would you describe your overall physical activity?

☐ Increased significantly

☐ Increased slightly

☐ Stayed the same

☐ Decreased slightly

☐ Decreased significantly

13.3 How did you first learn about walking football?

☐ Through a local football club

☐ From a district or regional football association

☐ Through a friend or family member

☐ Social media or other online platforms

☐ Media coverage (e.g., newspapers, TV)

☐ From the FA’s website or other official football websites

☐ Through a community event or local group

Other:____

(Please turn over for final page)

Future Plans

14.1 Do you plan to continue playing walking football in the long term (e.g., over the next year or more)?

☐ Yes, definitely ☐ Yes, likely ☐ Unsure ☐ No, unlikely ☐ No, definitely not

Please describe the main reasons for your decision:

____

14.2 Do you have any suggestions for improving walking football or ensuring your continued participation?

____

Thank you for taking the time to complete the questionnaire.
